# Device-measured physical activity and cardiac structure by magnetic resonance

**DOI:** 10.1093/eurheartj/ehae506

**Published:** 2024-08-14

**Authors:** Thomas Yates, Cameron Razieh, Joe Henson, Alex V Rowlands, Jonathan Goldney, Gaurav S Gulsin, Melanie J Davies, Kamlesh Khunti, Francesco Zaccardi, Gerry P McCann

**Affiliations:** Diabetes Research Centre, University of Leicester, Leicester General Hospital, Leicester LE5 4PW, UK; Leicester Diabetes Centre, University Hospitals of Leicester NHS Trust, Leicester LE5 4PW, UK; Diabetes Research Centre, University of Leicester, Leicester General Hospital, Leicester LE5 4PW, UK; Leicester Diabetes Centre, University Hospitals of Leicester NHS Trust, Leicester LE5 4PW, UK; Diabetes Research Centre, University of Leicester, Leicester General Hospital, Leicester LE5 4PW, UK; Leicester Diabetes Centre, University Hospitals of Leicester NHS Trust, Leicester LE5 4PW, UK; Diabetes Research Centre, University of Leicester, Leicester General Hospital, Leicester LE5 4PW, UK; Leicester Diabetes Centre, University Hospitals of Leicester NHS Trust, Leicester LE5 4PW, UK; Diabetes Research Centre, University of Leicester, Leicester General Hospital, Leicester LE5 4PW, UK; Leicester Diabetes Centre, University Hospitals of Leicester NHS Trust, Leicester LE5 4PW, UK; Department of Cardiovascular Sciences, University of Leicester, Leicester, UK; Diabetes Research Centre, University of Leicester, Leicester General Hospital, Leicester LE5 4PW, UK; Leicester Diabetes Centre, University Hospitals of Leicester NHS Trust, Leicester LE5 4PW, UK; Diabetes Research Centre, University of Leicester, Leicester General Hospital, Leicester LE5 4PW, UK; Leicester Diabetes Centre, University Hospitals of Leicester NHS Trust, Leicester LE5 4PW, UK; Leicester Real World Evidence Unit, University of Leicester, Leicester, UK; Diabetes Research Centre, University of Leicester, Leicester General Hospital, Leicester LE5 4PW, UK; Leicester Diabetes Centre, University Hospitals of Leicester NHS Trust, Leicester LE5 4PW, UK; Leicester Real World Evidence Unit, University of Leicester, Leicester, UK; Department of Cardiovascular Sciences, University of Leicester, Leicester, UK

**Keywords:** Accelerometer, Athletes heart, Cardiac magnetic resonance, Cardiac remodelling, Left ventricular end-diastolic volume, Left ventricular wall thickness, Physical activity

## Abstract

**Background and Aims:**

Although extreme cardiac adaptions mirroring phenotypes of cardiomyopathy have been observed in endurance athletes, adaptions to high levels of physical activity within the wider population are under-explored. Therefore, in this study, associations between device-measured physical activity and clinically relevant cardiac magnetic resonance volumetric indices were investigated.

**Methods:**

Individuals without known cardiovascular disease or hypertension were included from the UK Biobank. Cardiac magnetic resonance data were collected between 2015 and 2019, and measures of end-diastolic chamber volume, left ventricular (LV) wall thickness, and LV ejection fraction were extracted. Moderate-to-vigorous-intensity physical activity (MVPA), vigorous-intensity physical activity (VPA), and total physical activity were assessed via wrist-worn accelerometers.

**Results:**

A total of 5977 women (median age and MVPA: 62 years and 46.8 min/day, respectively) and 4134 men (64 years and 49.8 min/day, respectively) were included. Each additional 10 min/day of MVPA was associated with a 0.70 [95% confidence interval (CI): 0.62, 0.79] mL/m^2^ higher indexed LV end-diastolic volume (LVEDVi) in women and a 1.08 (95% CI: 0.95, 1.20) mL/m^2^ higher LVEDVi in men. However, even within the top decile of MVPA, LVEDVi values remained within the normal ranges [79.1 (95% CI: 78.3, 80.0) mL/m^2^ in women and 91.4 (95% CI: 90.1, 92.7) mL/m^2^ in men]. Associations with MVPA were also observed for the right ventricle and the left/right atria, with an inverse association observed for LV ejection fraction. Associations of MVPA with maximum or average LV wall thickness were not clinically meaningful. Results for total physical activity and VPA mirrored those for MVPA.

**Conclusions:**

High levels of device-measured physical activity were associated with cardiac remodelling within normal ranges.

## Structured Graphical Abstract

**Structured Graphical Abstract:**
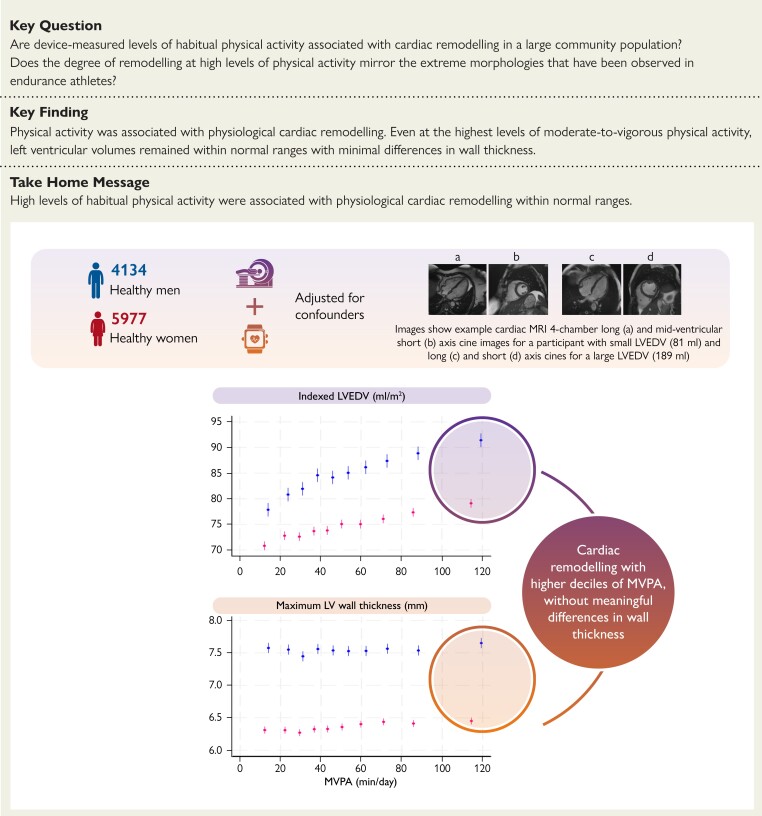
Association between moderate-to-vigorous-intensity physical activity and left ventricular parameters in women and men. LV chamber sizes were larger with higher physical activity, but remained within normal ranges, whilst LV wall size was not meaningfully different. LV, left ventricular; LVEDV, left ventricular end-diastolic volume; MRI, magnetic resonance imaging; MVPA, moderate-to-vigorous-intensity physical activity.

**See the editorial comment for this article ‘The challenge of interpreting cardiac adaptation to exercise: the importance of picking up the training history', by V. Maestrini *et al*., https://doi.org/10.1093/eurheartj/ehae535.**

## Introduction

Physical activity is a fundamental tool in the primary and secondary prevention of cardiovascular disease (CVD)^1^, whilst also representing a first-line treatment in the management of a number of modifiable cardiovascular risk factors (e.g. blood pressure, hypercholesterolaemia, and Type 2 diabetes mellitus).^2^ More broadly, higher levels of physical activity are associated with reduced rates of all-cause and cardiovascular-specific mortality (∼30%–40%).^3–5^

The cardiovascular benefits of physical activity may, in part, be mediated through the positive effect on cardiac structure and function. Regular physical activity subjects the heart to haemodynamic stresses which, in order to meet the systemic demand, undergoes morphological adaptations. The most widely studied of these is the left ventricle, where aerobic or endurance-based physical activity and exercise training have consistently been associated with a number of changes, such as a larger mass, greater posterior wall and interventricular septal thickness, and larger end-diastolic diameters or volumes.^6^ Structural adaptations to physical activity have also been shown in the right ventricle, through increases in volume and mass,^7–9^ and in the atria,^10^ where enlargement is typically proportional to left ventricular (LV) adaptations.^11^

Whilst moderate levels of physical activity and exercise training result in positive cardiac remodelling, there is an established phenomenon whereby high levels of endurance exercise result in extreme adaptions to LV volume, wall thickness, and mass that may overlap with diagnostic criteria and pathologies of dilated and hypertrophic cardiomyopathies,^12^ changes commonly referred to as the ‘athlete’s heart’.^13^ Such structural changes can make it difficult to clearly distinguish between physiological adaptions and pathological changes (i.e. ‘grey zone’).^14,15^ Although the clinical implications of extreme cardiac remodelling associated with an athlete’s heart are a matter of debate, the longer-term prognosis is largely unknown, with a paucity of high-quality evidence. This uncertainty reflects the wider literature where high levels of exercise or vigorous-intensity physical activity (VPA) have been shown to result in a loss of association with health outcomes or an increased risk at high volumes in some, but not all, studies.^5,16^

To date, much of the epidemiological work quantifying the association of physical activity with cardiac remodelling has been undertaken in elite or professional athletes participating in high levels of structured exercise, who represent a very small proportion of the wider population. Conversely, there is a paucity of data examining how the range of habitual physical activity contributes to cardiac remodelling and whether high levels of habitual physical activity within a more representative general population are also associated with extreme adaptations. The few studies that have been published provide some evidence that high levels of recreational physical activity may increase the risk of LV hypertrophy or extreme dilation,^7,8,17,18^ but findings are limited by small sample sizes, self-reported physical activity, or low numbers with high physical activity volumes.

Technological advancements in device-measured physical activity, coupled with cardiac magnetic resonance (CMR) imaging—the gold standard for the evaluation of global and regional cardiac structure and function,^19^ allow for more accurate and reproducible estimations in large cohorts. The aim of this study was to investigate the nature of the association between device-measured physical activity and cardiac remodelling in a large general population of healthy adults without CVD or hypertension.

## Methods

### Cohort definition

The UK Biobank is a large cohort study with data collected on a wide range of clinical, demographic, and lifestyle factors. The initial baseline visit occurred between March 2006 and July 2010 in more than 500 000 women and men recruited across England, Wales, and Scotland. An imaging sub-study was introduced in 2014, with participants invited to attend whole-body imaging protocols, which included CMR.^20^ In May 2023, valid CMR data, covering 39 697 participants between 2014 and 2019, were made available to investigators. Accelerometer data were collected in a random sub-cohort of participants with valid email addresses overlapping the scanning sub-study (from 2013 to 2015).^21^ In total, 14 680 participants had combined CMR and accelerometer data available, with complete information on covariables. Using data from the scanning visit or the nearest preceding assessment, we excluded from this initial cohort individuals with CVD or diagnosed hypertension or taking commonly prescribed blood pressure medications (angiotensin receptor blockers, angiotensin-converting enzyme inhibitors, beta-blockers, calcium channel blockers) (definitions for all criteria are displayed in Supplementary data online, *Figure S1*), leaving 10 111 with complete data available for analysis (see Supplementary data online, *Figure S1*).

This analysis was performed as part of UK Biobank project number 33266. Ethical approval for the UK Biobank was obtained from the North West Research Ethics Committee (reference number: 11/NW/0382, 16/NW/0274), and in Scotland, the UK Biobank received ethical approval from the Community Health Index Advisory Group. This study complied with the Declaration of Helsinki, and written informed consent was obtained from all participants.

### Cardiac magnetic resonance

Cardiac magnetic resonance scans were performed on a 1.5-T scanner (MAGNETOM Aera, Syngo Platform VD13A, Siemens Healthcare, Erlangen, Germany) following a standardized protocol published previously.^22^ An assessment of the left and right ventricles was conducted using a complete short-axis stack of balanced steady-state free precession sequences. Left ventricular papillary muscles were included in end-diastolic volume assessments but excluded from LV mass assessments. The left and right atria were segmented on long-axis four-chamber-view (4Ch) cine images. Left atrial measurements were also derived from the vertical long-axis (two-chamber) view, and left atrial volumes were calculated according to the biplane area–length method. Left ventricular wall thickness was measured in each image slice (categorized and averaged across established American Heart Association segments^23^) using the distance between the endocardial contour and the epicardial contour at the end-diastolic frame. All image segmentations were manually quality-controlled by a trained cardiologist, with images showing poor segmentations, insufficient LV coverage, or missing anatomical structures discarded. Images were analysed using cvi42 post-processing software (version 5.1.1, Circle Cardiovascular Imaging Inc., Calgary, Canada) and employing standard operating procedures developed and approved prior to study commencement. We extracted the following information for analysis: LV end-diastolic volume (LVEDV), LV mass, LV wall thickness for each segment (with the maximum and mean LV wall thickness values calculated), LV ejection fraction, right ventricular end-diastolic volume (RVEDV), maximum left and right atrial volumes, and resting heart rate during the scan. A measure of cardiac remodelling, referred to as concentricity, was derived as LV mass/LVEDV. LVEDV, LV mass, and RVEDV were indexed (denoted in variable names by ‘i’) through dividing by body surface area (BSA), which was calculated using the DuBois’ equation:^24^ BSA (m^2^) = weight (kg)^0.425^ × height (cm)^0.725^ × 0.007184. Based on the previous definition used for CMR,^25,26^ maximum wall thickness values <11 mm were considered normal, with values of ≥11 mm categorized as mild-to-severe hypertrophy. LVESV, RVESV, and minimum left and right atrial volumes were also extracted and reported for descriptive purposes.

### Accelerometer analysis

Accelerometer data (in 5-s epochs) were downloaded from the UK Biobank. The files were converted to R-format for entry into R-package GGIR version 1.10-7 (http://cran.r-project.org). Files with fewer than 3 days of data (defined as >16 h/day of valid wear data) or where data were not available for each 15-min period of the 24-h cycle were excluded, as previously described.^27,28^ Files were also excluded if they failed calibration criteria [post-calibration error >0.01 g (10 m*g*)]. Time spent in moderate-to-vigorous-intensity physical activity (MVPA) in bouts of ≥1 min was calculated using a threshold of 100 m*g.*^29^ Time in VPA in bouts of ≥1 min was further calculated using a threshold of 400 m*g*.^29^ Moderate-to-vigorous-intensity physical activity and VPA accumulated in bouts with a minimum duration of 1 min were used to identify physical activity that involved an aerobic component, removing incidental or anaerobic physical activity. Average acceleration across the 24-h day is reported in milligravity (m*g*) units, quantified by the Euclidean Norm Minus One method, used to describe the total level of physical activity (volume) undertaken.^30^ Accelerometer outcomes were averaged across valid days.

### Covariables

Information was extracted on the following potential confounders of the association between physical activity and cardiac structure: sex, age (at imaging visit), height, weight, ethnicity (White European, South Asian, Black, other), deprivation (using the Townsend index score, a composite of four domains: unemployment, non-car ownership, non-home ownership, and household overcrowding using census data at the postcode level), smoking status (current, previous, never), diabetes status (yes, no), statin medication status (yes, no), family history of CVD (parental; yes, no), family history of hypertension (parental; yes, no), and systolic blood pressure. Covariate data were obtained from the imaging visit where available or from the nearest preceding assessment. Codes for the included covariates are listed in Supplementary data online, *Figure S1*.

### Statistical analysis

For each CMR outcome variable, sex-stratified linear regression models with MVPA as the exposure (independent variable) were adjusted for number of accelerometer wear days, season of accelerometer wear (using two orthogonal sine functions, described previously^31^), age, ethnicity, deprivation, smoking, diabetes, statin treatment, family history of CVD, and family history of hypertension; non-indexed outcome variables were additionally adjusted for BSA (Model 1). Resting heart rate during the magnetic resonance imaging scan was included as an additional outcome to help interpret CMR outcomes, particularly ejection fraction.

Using the Bayesian information criterion (BIC), for each CMR outcome, we compared Model 1 with a linear vs. spline transformation of MVPA, with a number of knots ranging from 3 to 7 located at centiles of MVPA as suggested by Harrell;^32^ linear models consistently displayed the best fit and were therefore selected for the analyses. To graphically display the shape of the associations, we estimated the marginal means for each outcome across deciles of MVPA and plotted them against the within-decile MVPA medians. A second linear regression model was additionally adjusted for systolic blood pressure (Model 2), which was added separately as it was hypothesized to potentially mediate, rather than confound, the association between physical activity and cardiac structure.

As CMR outcomes are known to vary by age,^33,34^ we further assessed whether the pattern of association between MVPA and CMR outcomes was consistent across age; this was undertaken by adding an age by MVPA interaction term to Model 1. For each outcome, models with the added interaction term consistently displayed a poorer model fit (higher BIC), suggesting that associations were not modified by age. In order to display these results in a sensitivity analysis, we plotted the predicted marginal means for the association between deciles of MVPA and CMR outcomes for ages 50, 60, and 70 years.

Moderate-to-vigorous-intensity physical activity was selected as the main exposure of interest, with analysis for LV parameters repeated across total physical activity and VPA in order to assess whether the pattern of association was affected by the exposure of physical activity selected.

All analyses were performed in Stata BE version 18, and results were reported with 95% confidence interval (CI); a *P*-value <.05 was considered statistically significant.

## Results

The analysis included 5977 women (median age and MVPA: 62 years and 46.8 min/day, respectively) and 4134 men (64 years and 49.8 min/day, respectively); sex-stratified cohort characteristics are reported in *Table 1*.

**Table 1:** Cohort characteristics

	Women	Men	Total
No. of participants	5977	4134	10 111
Age at imaging visit (years)	62 (56, 68)	64 (57, 69)	63 (56, 68)
Ethnic group			
White European	5881 (98.4)	4053 (98.0)	9934 (98.2)
South Asian	24 (0.4)	36 (0.9)	60 (0.6)
Black African or Caribbean	24 (0.4)	22 (0.5)	46 (0.5)
Other	48 (0.8)	23 (0.6)	71 (0.7)
Townsend deprivation index	−2.7 (−3.9, −0.6)	−2.7 (−3.9, −0.6)	−2.7 (−3.9, −0.6)
Smoking status			
Never	4008 (67.1)	2587 (62.6)	6595 (65.2)
Previous	1806 (30.2)	1371 (33.2)	3177 (31.4)
Current	163 (2.7)	176 (4.3)	339 (3.4)
Statin medication use	396 (6.6)	628 (15.2)	1024 (10.1)
Diabetes	82 (1.4)	101 (2.4)	183 (1.8)
Family history of cardiovascular disease	3296 (55.1)	2108 (51.0)	5404 (53.4)
Family history of hypertension	2736 (45.8)	1524 (36.9)	4260 (42.1)
Systolic blood pressure (mmHg)	126.0 (116.5, 136.5)	133.5 (125.0, 143.0)	129.0 (119.5, 140.0)
Body surface area (m^2^)	1.7 (1.6, 1.8)	2.0 (1.9, 2.1)	1.8 (1.7, 2.0)
Average heart rate during CMR scan (beats/min)	62.0 (56.0, 69.0)	59.0 (53.0, 66.0)	61.0 (55.0, 68.0)
LV end-diastolic volume (mL)	127.1 (113.4, 142.6)	166.1 (147.3, 186.7)	140.5 (121.4, 164.6)
LV end-systolic volume (mL)	49.1 (42.1, 57.1)	69.3 (59.1, 81.4)	56.0 (46.1, 68.7)
Indexed LV end-diastolic volume (mL/m^2^)	74.2 (67.3, 81.8)	84.3 (75.4, 93.8)	77.7 (69.7, 87.0)
Indexed LV end-systolic volume (mL/m^2^)	28.7 (24.8, 32.9)	35.2 (30.2, 40.7)	30.9 (26.5, 36.4)
LV ejection fraction (%)	61.1 (57.5, 64.7)	58.0 (54.4, 61.8)	59.9 (56.2, 63.7)
LV myocardial mass (g)	68.2 (61.4, 76.1)	98.7 (88.5, 110.5)	78.1 (66.0, 95.5)
LV mean wall thickness (mm)	5.0 (4.7, 5.4)	6.0 (5.6, 6.4)	5.4 (4.9, 5.9)
LV maximum wall thickness (mm)	6.3 (5.9, 6.8)	7.5 (7.0, 8.0)	6.7 (6.1, 7.5)
Concentricity (g/mL)	0.5 (0.5, 0.6)	0.6 (0.5, 0.6)	0.6 (0.5, 0.6)
RV end-diastolic volume (mL)	132.4 (118.0, 148.8)	180.9 (159.5, 203.7)	148.5 (127.1, 177.2)
RV end-systolic volume (mL)	53.9 (45.9, 63.3)	80.6 (68.5, 94.3)	63.1 (50.9, 78.7)
Indexed RV end-diastolic volume (mL/m^2^)	77.2 (69.9, 85.4)	92.0 (81.9, 101.9)	82.2 (73.2, 93.3)
Indexed RV end-systolic volume (mL/m^2^)	31.4 (27.2, 36.3)	40.9 (35.2, 47.4)	34.8 (29.4, 41.6)
Left atrium maximum volume (mL)	65.4 (53.7, 77.7)	73.9 (59.2, 90.8)	68.2 (55.8, 82.7)
Left atrium minimum volume (mL)	24.8 (18.7, 31.6)	28.7 (20.8, 37.5)	26.3 (19.4, 33.8)
Indexed left atrium maximum volume (mL/m^2^)	37.9 (31.5, 44.9)	37.3 (30.3, 45.8)	37.7 (31.0, 45.1)
Indexed left atrium minimum volume (mL/m^2^)	14.4 (10.9, 18.3)	14.5 (10.6, 18.9)	14.5 (10.8, 18.5)
Right atrium maximum volume (mL)	75.0 (63.6, 87.8)	96.7 (79.8, 117.9)	82.2 (68.2, 100.3)
Right atrium minimum volume (mL)	37.9 (30.8, 46.2)	53.9 (42.6, 67.0)	43.0 (33.9, 55.2)
Indexed right atrium maximum volume (mL/m^2^)	43.7 (37.0, 50.8)	48.9 (40.1, 59.4)	45.4 (38.1, 54.1)
Indexed right atrium minimum volume (mL/m^2^)	21.9 (17.0, 26.6)	27.2 (31.3, 33.8)	23.7 (19.0, 29.6)
Moderate-to-vigorous-intensity physical activity (min/day)	46.8 (29.5, 71.0)	49.8 (31.0, 72.8)	47.9 (30.1, 71.7)
Vigorous-intensity physical activity (min/day)	0.0 (0.0, 0.9)	0.2 (0.0, 1.4)	0.2 (0.0, 1.1)
Total physical activity (m*g*)	29.1 (24.5, 34.5)	28.4 (23.5, 34.2)	28.8 (24.1, 34.4)
Valid days of accelerometer wear			
3	100 (1.7)	85 (2.1)	185 (1.8)
4	232 (3.9)	161 (3.9)	393 (3.9)
5	482 (8.1)	282 (6.8)	764 (7.6)
6	5163 (86.4)	3606 (87.2)	8769 (86.7)

Shown are median (interquartile range) or number (column-wise percentage).

A greater Townsend index score indicates a greater degree of deprivation.

Covariable data were obtained from the scanning visit where available or from the nearest preceding assessment.

The distributions for MVPA, VPA, and total physical activity are reported in Supplementary data online, *Table S1*. There was a wide distribution in MVPA: in women, values ranged from 0 to 17.7 min/day in the bottom decile and 97.6 to 288.4 min/day in the top decile; the corresponding values for men were 0.3–19.3 and 99.2–240.2 min/day. Levels of VPA were lower, reaching a range of 4.9–70.6 min/day in the top decile for women and 7.0–80.2 min/day in the top decile for men.

**Figure 1:**
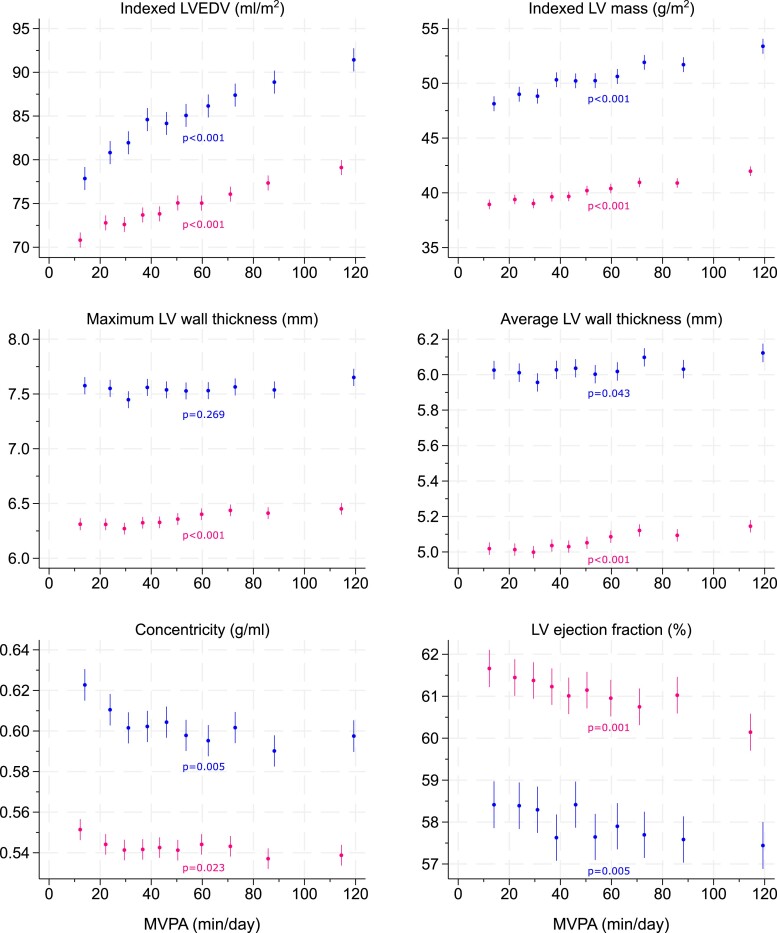
An association between moderate-to-vigorous-intensity physical activity and left ventricular parameters. Values of moderate-to-vigorous-intensity physical activity are sex-specific within-decile medians; the bars indicate 95% confidence intervals. Blue, men; pink, women. The *P*-values show the linear trend adjusted for accelerometer wear duration, season, age, ethnicity, deprivation, smoking status, diabetes, statin medication, family history of cardiovascular disease, and family history of hypertension. Wall thickness outcomes, concentricity, and ejection fraction models additionally adjusted for body surface area

**Figure 2:**
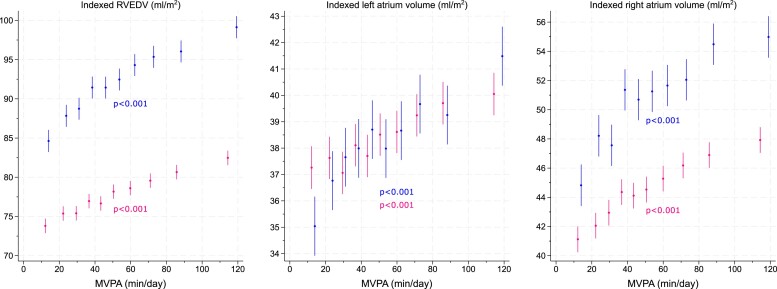
An association between moderate-to-vigorous-intensity physical activity and right ventricular, left atrial, and right atrial end-diastolic volumes. Values of moderate-to-vigorous-intensity physical activity are sex-specific within-decile medians; the bars indicate 95% confidence intervals. Blue, men; pink, women. The *P*-values show the linear trend adjusted for accelerometer wear duration, season, age, ethnicity, deprivation, smoking status, diabetes, statin medication, family history of cardiovascular disease, and family history of hypertension

*Figure 1* shows the association between deciles of MVPA and LV outcomes in women and men, whilst *Table 2* reports the linear associations per 10 min/day higher MVPA. There was a strong and linear dose–response association between MVPA and LVEDVi: for every 10 min of MVPA, LVEDVi was 0.70 (95% CI: 0.62, 0.79) mL/m^2^ higher in women and 1.08 (95% CI: 0.95, 1.20) mL/m^2^ higher in men, reaching a value of 79.1 (95% CI: 78.3, 80.0) mL/m^2^ in the top decile of MVPA for women and 91.4 (95% CI: 90.1, 92.7) mL/m^2^ in the top decile of MVPA for men (*Figure 1*). Positive and linear associations between MVPA and chamber volumes were also observed for RVEDVi and maximum left and right atrial volumes (*Figure 2* and *Table 2*).

**Table 2:** Multivariable associations between physical activity and cardiac magnetic resonance image indices

Sex	Outcome	Linear association per 10 min/day difference in MVPA	*P*-value
Women	Indexed LVEDV (mL/m^2^)	0.703 (0.621, 0.786)	<.001
	Indexed LV mass (g/m^2^)	0.276 (0.234, 0.318)	<.001
	Maximum LV wall thickness (mm)	0.016 (0.011, 0.021)	<.001
	Average LV wall thickness (mm)	0.013 (0.010, 0.017)	<.001
	Concentricity (g/mL)	−0.001 (−0.001, −0.000)	.003
	LV ejection fraction (%)	−0.123 (−0.167, −0.080)	<.001
	Indexed RVEDV (mL/m^2^)	0.759 (0.671, 0.848)	<.001
	Indexed left atrium volume (mL/m^2^)	0.270 (0.191, 0.348)	<.001
	Indexed right atrium volume (mL/m^2^)	0.578 (0.492, 0.663)	<.001
Men	Indexed LVEDV (mL/m^2^)	1.075 (0.947, 1.203)	<.001
	Indexed LV mass (g/m^2^)	0.431 (0.365, 0.497)	<.001
	Maximum LV wall thickness (mm)	0.008 (0.001, 0.016)	.033
	Average LV wall thickness (mm)	0.010 (0.005, 0.015)	.002
	Concentricity (g/mL)	−0.002 (−0.003, −0.001)	<.001
	LV ejection fraction (%)	−0.092 (−0.147, −0.038)	.001
	Indexed RVEDV (mL/m^2^)	1.181 (1.045, 1.317)	<.001
	Indexed left atrium volume (mL/m^2^)	0.455 (0.346, 0.563)	<.001
	Indexed right atrium volume (mL/m^2^)	0.788 (0.650, 0.927)	<.001

Data are reported as a coefficient (95% CI).

Coefficients adjusted for wear duration, season, age, ethnicity, deprivation, smoking status, diabetes, statin medication, family history of CVD, and family history of hypertension. Wall thickness outcomes, concentricity, and ejection fraction models additionally adjusted for body surface area.

**Figure 3:**
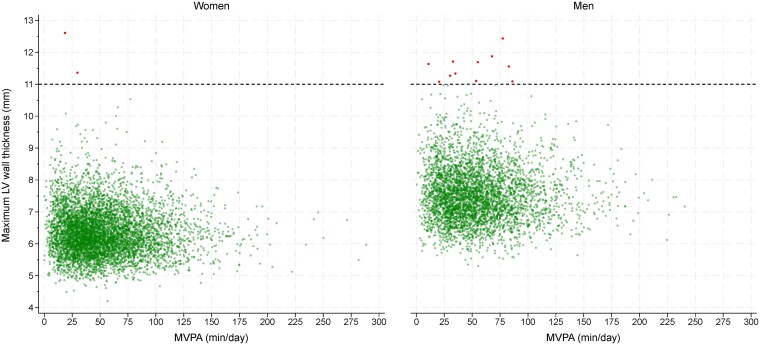
Distribution of moderate-to-vigorous-intensity physical activity and maximum wall thickness values. The dashed line shows the threshold used to define mild-to-severe hypertrophy

There were also associations between MVPA and LV mass in men and women, but to a lesser extent than LVEDVi, resulting in lower concentricity values with higher MVPA (*Figure 1* and *Table 2*). Although there was an association between MVPA and maximum and average wall thickness in women, differences were small as each additional 10 min of MVPA was associated with a difference of 0.02 (95% CI: 0.01, 0.02) mm in maximum wall thickness and of 0.01 (95% CI: 0.01, 0.02) mm in average wall thickness (*Table 2*); consequently, maximum wall thickness values ranged from 6.3 (95% CI: 6.3, 6.4) mm in the bottom decile of MVPA to 6.5 (95% CI: 6.4, 6.5) mm in the top decile of MVPA (*Figure 1*). Men followed a similar pattern of association, with maximum wall thickness ranging from 7.6 (95% CI: 7.5, 7.7) mm in the bottom decile of MVPA to 7.7 (95% CI: 7.6, 7.7) mm in the top decile of MVPA (*Figure 1* and *Table 2*). There were 13 cases of mild-to-severe hypertrophy (2 in women and 11 in men, all with thickness ≥11 mm), with maximum wall thickness values tending to cluster within the lower distribution of MVPA (*Figure 3*).

There was evidence that higher levels of MVPA were associated with lower resting LV ejection fractions (*Figure 1*); this result was in the context of a corresponding small inverse association between MVPA and resting heart rate (see Supplementary data online, *Figure S2*).

Although older age was associated with smaller LVEDVi values and greater wall thicknesses, particularly in women, the pattern of association between MVPA and LV volumetric and wall thickness indices was consistent across ages (see Supplementary data online, *Figure S3*).

Associations between CMR indices and MVPA were not affected by further adjustment for systolic blood pressure (see Supplementary data online, *Table S2*).

Results for total physical activity and VPA followed the same pattern of association as those observed for MVPA (see Supplementary data online, *Figures S4* and *S5*): the top decile of VPA was associated with an LVEDVi size of 79.2 (95% CI: 78.3, 80.0) mL/m^2^ in women and 95.4 (95% CI: 94.1, 96.8) mL/m^2^ in men but without evidence of meaningful associations with wall thickness.

## Discussion

Device-measured physical activity was associated with greater LVEDVi, indicative of eccentric remodelling, with proportional associations for RVEDVi and maximal left and right atrial volumes. However, even at the highest levels of physical activity, the observed levels of LVEDVi were within the normal ranges obtained from independent cohorts along with those reported for the UK Biobank,^33,35,36^ and there was no indication that higher levels of physical activity were associated with meaningful differences in LV wall thickness. Therefore, even at the highest levels of physical activity undertaken within a large general population of healthy adults—with values ranging from 98 to 288 min/day in the top decile of MVPA—there was no evidence that the degree of cardiac remodelling overlapped with LV volumetric or wall thickness values consistent with dilated or hypertrophic cardiomyopathies (*Structured Graphical Abstract*).

This study is the largest to report associations between device-measured physical activity and CMR metrics of cardiac structure and function, with our findings extending the few smaller studies reported previously. An earlier preliminary study of 1030 participants from the UK Biobank found no association between total physical activity and LV trabeculation, although associations with other clinically relevant measures of wall thickness and end-diastolic volumes were not reported.^37^ Our findings are somewhat in contrast to the Dallas Heart Study which, in a sample of 1368 participants free from CVD, suggested that remodelling associated with VPA overlapped with CMR-defined malignant LV hypertrophy.^17^ Similarly, the Multi-Ethnic Study of Atherosclerosis reported that high levels of self-reported physical activity were associated with eccentric LV hypertrophy in 2779 participants,^18^ with the UK Digital Heart Project in 1096 participants also finding regular self-reported exercise or competitive sports increased the risk of a dilated left ventricle outside of normal ranges.^8^ However, there are important differences in these previous studies compared with the present one. Notably, there were less extensive exclusions for past medical history and the prevalence of LV hypertrophy was high in all three previous studies, ranging from 3% to 15%,^8,17,18^ with criteria based on LV volumetric or mass thresholds derived from population distributions in small single studies rather than clinical criteria. In contrast, the prevalence of hypertrophy based on established LV wall thickness thresholds within the wider UK Biobank imaging cohort has been reported at 0.22% in men and 0.04% in women, which is consistent with the wider literature.^38,39^ As such, it is difficult to directly compare the present study to the previous literature.

When placed in the context of LV wall thickness values or the latest pooled evidence on normal ranges for LVEDVi, our study did not find evidence of clinically meaningful adaptions to cardiac structure in association with high physical activity. For example, although women and men in the top decile within our larger UK Biobank population undertook over 11 h/week of MVPA, the upper 95% CI of LVEDVi in this decile was ≤80 mL/m^2^ in women and <93 mL/m^2^ in men, which are below the thresholds of 96 mL/m^2^ for women and 108 mL/m^2^ for men that have been used to define the upper limit of normal using previous CMR studies.^36^ Importantly, the higher LVEDVi values with higher MVPA were not accompanied by meaningful adaptions to LV wall thickness, further suggesting eccentric remodelling. The maximal wall thickness values in those within the top decile of MVPA were 6.5 (95% CI: 6.4, 6.5) mm in women and 7.7 (95% CI: 7.6, 7.7) mm in men, substantially below the threshold of 11 mm used to identify mild-to-severe hypertrophy, with the highest wall thickness values tending to cluster within the lower distribution of MVPA; none of the participants in the highest decile of MVPA had mild-to-severe hypertrophy.

We also report an inverse association between MVPA and ejection fraction. Although a reduction in LV ejection fraction has been observed for elite athletes,^40^ evidence for habitual levels of physical activity is sparse with inconsistent findings.^8,18^ We hypothesize our results are due to the strong dose–response association between MVPA and LVEDVi which, through not being fully offset by the less pronounced inverse association between MVPA and resting heart rate, required a lower ejection fraction at higher LVEDVi to maintain homeostasis. Although ejection fraction values were lower with higher MVPA, the lower 95% CI values corresponding to the highest decile of MVPA remained above an LV ejection value of 55% (indicating normal function).

Results stratified by sex and specifically ages revealed greater LVEDVi and LV wall thickness values in men than in women, whereas older age was associated with smaller LVEDVi and greater wall thickness values, particularly in women. These sex and age differences are consistent with the wider literature.^33–36^ However, despite these differences in cardiac structure, associations between MVPA and CMR outcomes were notable for their consistency across sex and age. Therefore, the conclusions of dose–response eccentric remodelling within normal ranges appeared to be generalizable to women and men along with old and young alike.

This study is strengthened by the large sample with four-chamber CMR-derived metrics of cardiac structure and function, combined with device-measured physical activity. Limitations include the observational cross-sectional design that precludes inferences of causality and the single 7-day measurement period of physical activity, which may act to dilute associations with CMR indices, including those indicative of hypertrophy or extreme dilation where longer-term chronic exposures to high levels of physical activity may be important. However, it has previously been shown that objectively measured MVPA and total physical activity have reasonable reproducibility over time at a population level,^41^ with a 7-day measurement period consequently reflecting longer-term habitual activity. Furthermore, wrist-worn accelerometers primarily quantify levels of ambulatory-based activity; therefore, aerobic physical activities with limited wrist movement, such as cycling, may not have been fully captured. There are also some potential limitations with the automated CMR analysis pipeline, which may be less accurate when analysing scans with uncommon features. However, quality control for image segmentation within the UK Biobank was performed manually. Differences in the assessment of the left and right atria (biplane vs. single plane) mean that any differences in the association with physical activity should not be over-interpreted. The characteristics of the UK Biobank population are both a strength and a limitation in the context of this study: the UK Biobank participants are healthier than the UK general population,^42^ with the further exclusions for pre-existing CVD and hypertension in this study making confounding due to underlying chronic disease less likely than in a general population. Although the healthy nature of the cohort also coincided with high levels of MVPA, even in this physically active population, levels of VPA were low and outside the range of recreational endurance athletes. Therefore, there is potential for a lack of generalizability to general populations with a higher burden of underlying health conditions or those undertaking extreme levels of VPA. Finally, as 98.2% of the cohort were White European, results may not be generalizable to ethnic minorities, which is important, given that ethnic differences in cardiac structure and function have been observed previously,^43,44^ with profound electrical and structural alterations in response to exercise training noted in athletes of African or Afro-Caribbean ethnicity.^45^

## Conclusions

The range of device-assessed physical activity undertaken within a large healthy adult community population was associated with dose–response eccentric remodelling within normal ranges without clinically meaningful differences in wall thickness. Therefore, if a threshold exists beyond which the risk of LV hypertrophy or extreme dilation occurs, it is likely to be outside the range of physical activity undertaken within this population. Given the relative paucity of data in the general populations, of which this study is the largest to date, and the inconsistent findings from the few that have been published, further investigation with cohorts enriched by very high levels of physical activity is required.

### Open science and replicability

The list of codes used to define the population is reported in Supplementary data online, *Figure S1*.

## Authors’ contributions

T.Y., G.P.M., J.H., C.R., and J.G. formed the core working group and developed the research question. F.Z., C.R., and T.Y. developed the analysis plan and code, with C.R. preparing the analysis data set. T.Y. and J.H. drafted the manuscript. All authors contributed to the interpretation and revised the manuscript for important intellectual content.

## Supplementary Material

ehae506_Supplementary_Data

## Data Availability

The UK Biobank resource can be accessed by researchers on application at https://www.ukbiobank.ac.uk/. This analyses was conducted under application #33266.
