# Supplementary material for: Device-measured physical activity and cardiac structure by magnetic resonance
Source: Eur Heart J. 2024 Aug 14;46(2):176–86. doi: 10.1093/eurheartj/ehae506 (PMC11704417; doi:10.1093/eurheartj/ehae506)
Supplement: ehae506_Supplementary_Data [file ehae506_supplementary_data.docx]

**SUPPLEMENTARY MATERIAL**

Contents

[**Table S1:** Decile values for included physical activity exposures 2](#_Toc170457322)

[**Table S2:** Marginal means of CMR indices at deciles of moderate-vigorous physical activity 3](#_Toc170457323)

[**Figure S1:** Flow of cohort selection 4](#_Toc170457324)

[**Figure S2:** Association between MVPA and heart rate during the scan 5](#_Toc170457325)

[**Figure S3:** Association between MVPA and left ventricle parameters at ages 50, 60 and 70 years 6](#_Toc170457326)

[**Figure S4:** Association between total physical activity and left ventricle parameters 7](#_Toc170457327)

[**Figure S5:** Association between vigorous-intensity physical activity and left ventricle parameters 8](#_Toc170457328)

# **Table S1:** Decile values for included physical activity exposures

|  |  |  | **Moderate-vigorous**  **physical activity (min/day)** | | | |  | **Total**  **physical activity (mg)** | | | |  | **Vigorous**  **physical activity (min/day)** | | | |
| --- | --- | --- | --- | --- | --- | --- | --- | --- | --- | --- | --- | --- | --- | --- | --- | --- |
| **Sex** | **Decile** |  | **N** | **Median** | **Mean** | **Range** |  | **N** | **Median** | **Mean** | **Range** |  | **N** | **Median** | **Mean** | **Range** |
| Women | 1 |  | 598 | 12.2 | 11.7 | 0.0-17.7 |  | 598 | 19.1 | 18.5 | 8.9-21.0 |  | 3151 | 0 | 0 | 0-0 |
|  | 2 |  | 598 | 22.2 | 22.1 | 17.7-26.0 |  | 598 | 22.4 | 22.4 | 21.0-23.6 |  | - | - | - | - |
|  | 3 |  | 598 | 29.5 | 29.6 | 26.0-33.2 |  | 598 | 24.5 | 24.5 | 23.6-25.5 |  | - | - | - | - |
|  | 4 |  | 597 | 36.7 | 36.6 | 33.2-40.2 |  | 597 | 26.4 | 26.4 | 25.5-27.3 |  | - | - | - | - |
|  | 5 |  | 598 | 43.3 | 43.3 | 40.2-46.8 |  | 598 | 28.2 | 28.2 | 27.3-29.1 |  | - | - | - | - |
|  | 6 |  | 598 | 50.4 | 50.6 | 46.8-54.7 |  | 598 | 30.0 | 30.0 | 29.1-30.9 |  | 443 | 0.2 | 0.2 | 0.2-0.2 |
|  | 7 |  | 597 | 59.8 | 59.7 | 54.7-64.7 |  | 597 | 32.0 | 32.0 | 30.9-33.1 |  | 590 | 0.4 | 0.4 | 0.2-0.6 |
|  | 8 |  | 598 | 71.0 | 70.9 | 64.8-77.3 |  | 598 | 34.5 | 34.5 | 33.1-36.0 |  | 598 | 0.9 | 1.0 | 0.6-1.6 |
|  | 9 |  | 598 | 85.8 | 86.3 | 77.3-97.6 |  | 598 | 37.8 | 37.9 | 36.0-40.4 |  | 598 | 2.7 | 2.8 | 1.6-4.8 |
|  | 10 |  | 597 | 114.4 | 123.5 | 97.6-288.4 |  | 597 | 44.5 | 46.4 | 40.4-88.9 |  | 597 | 10.0 | 12.8 | 4.9-70.6 |
| Men | 1 |  | 415 | 14.0 | 13.2 | 0.3-19.3 |  | 414 | 17.6 | 17.3 | 7.9-19.6 |  | 1722 | 0 | 0 | 0-0 |
|  | 2 |  | 412 | 23.9 | 23.6 | 19.3-27.6 |  | 413 | 21.1 | 21.1 | 19.7-22.3 |  | - | - | - | - |
|  | 3 |  | 414 | 31.0 | 31.2 | 27.6-34.9 |  | 414 | 23.5 | 23.4 | 22.4-24.4 |  | - | - | - | - |
|  | 4 |  | 413 | 38.5 | 38.5 | 34.9-42.1 |  | 413 | 25.4 | 25.4 | 24.4-26.4 |  | - | - | - | - |
|  | 5 |  | 413 | 46.0 | 46.0 | 42.1-49.7 |  | 413 | 27.4 | 27.4 | 26.4-28.4 |  | 372 | 0.2 | 0.2 | 0.2-0.2 |
|  | 6 |  | 414 | 53.6 | 53.7 | 49.8-57.5 |  | 414 | 29.3 | 29.3 | 28.4-30.3 |  | 387 | 0.3 | 0.4 | 0.2-0.5 |
|  | 7 |  | 413 | 62.3 | 62.2 | 57.5-67.5 |  | 413 | 31.4 | 31.5 | 30.3-32.7 |  | 421 | 0.7 | 0.7 | 0.5-1.0 |
|  | 8 |  | 414 | 72.9 | 73.0 | 67.5-79.6 |  | 414 | 34.2 | 34.2 | 32.7-35.9 |  | 407 | 1.4 | 1.5 | 1.0-2.1 |
|  | 9 |  | 413 | 88.2 | 88.5 | 79.6-99.2 |  | 413 | 38.1 | 38.2 | 35.9-40.9 |  | 412 | 3.5 | 3.8 | 2.1-7.0 |
|  | 10 |  | 413 | 119.3 | 126.4 | 99.2-240.2 |  | 413 | 45.7 | 48.6 | 41.0-134.0 |  | 413 | 13.8 | 17.5 | 7.0-80.2 |

# **Table S2:** Marginal means of CMR indices at deciles of moderate-vigorous physical activity

| **Outcome** | **Model** | **Decile 1** | **Decile 2** | **Decile 3** | **Decile 4** | **Decile 5** | **Decile 5** | **Decile 7** | **Decile 8** | **Decile 9** | **Decile 10** |
| --- | --- | --- | --- | --- | --- | --- | --- | --- | --- | --- | --- |
|  | **Women** | | | | | | | | | | |
| **Indexed LVEDV (ml/m^2^)** | 1 | 70.8 (70.0, 71.7) | 72.8 (71.9, 73.6) | 72.6 (71.8, 73.5) | 73.7 (72.8, 74.5) | 73.8 (73.0, 74.7) | 75.1 (74.2, 75.9) | 75.0 (74.2, 75.9) | 76.1 (75.2, 76.9) | 77.3 (76.5, 78.2) | 79.1 (78.3, 80.0) |
|  | 2 | 70.8 (69.9, 71.6) | 72.7 (71.9, 73.6) | 72.6 (71.8, 73.5) | 73.7 (72.8, 74.5) | 73.8 (73.0, 74.7) | 75.1 (74.2, 75.9) | 75.1 (74.3, 76.0) | 76.1 (75.2, 76.9) | 77.4 (76.5, 78.2) | 79.1 (78.2, 79.9) |
| **Indexed LV mass (g/m^2^)** | 1 | 38.9 (38.5, 39.4) | 39.4 (39.0, 39.8) | 39.0 (38.6, 39.5) | 39.6 (39.2, 40.1) | 39.7 (39.2, 40.1) | 40.2 (39.8, 40.6) | 40.4 (40.0, 40.8) | 41.0 (40.5, 41.4) | 40.9 (40.5, 41.3) | 42.0 (41.5, 42.4) |
|  | 2 | 38.8 (38.4, 39.2) | 39.3 (38.9, 39.7) | 39.1 (38.7, 39.5) | 39.6 (39.1, 40.0) | 39.7 (39.3, 40.1) | 40.3 (39.8, 40.7) | 40.5 (40.1, 40.9) | 40.9 (40.5, 41.3) | 41.0 (40.6, 41.4) | 41.9 (41.5, 42.4) |
| **Maximum LV wall thickness (mm)** | 1 | 6.3 (6.3, 6.4) | 6.3 (6.3, 6.4) | 6.3 (6.2, 6.3) | 6.3 (6.3, 6.4) | 6.3 (6.3, 6.4) | 6.4 (6.3, 6.4) | 6.4 (6.3, 6.5) | 6.4 (6.4, 6.5) | 6.4 (6.4, 6.5) | 6.5 (6.4, 6.5) |
|  | 2 | 6.3 (6.3, 6.4) | 6.3 (6.2, 6.3) | 6.3 (6.2, 6.3) | 6.3 (6.3, 6.4) | 6.3 (6.3, 6.4) | 6.4 (6.3, 6.4) | 6.4 (6.4, 6.5) | 6.4 (6.4, 6.5) | 6.4 (6.4, 6.5) | 6.4 (6.4, 6.5) |
| **Average LV wall thickness (mm)** | 1 | 5.0 (5.0, 5.1) | 5.0 (5.0, 5.0) | 5.0 (5.0, 5.0) | 5.0 (5.0, 5.1) | 5.0 (5.0, 5.1) | 5.1 (5.0, 5.1) | 5.1 (5.1, 5.1) | 5.1 (5.1, 5.2) | 5.1 (5.1, 5.1) | 5.1 (5.1, 5.2) |
|  | 2 | 5.0 (5.0, 5.0) | 5.0 (5.0, 5.0) | 5.0 (5.0, 5.0) | 5.0 (5.0, 5.1) | 5.0 (5.0, 5.1) | 5.1 (5.0, 5.1) | 5.1 (5.1, 5.1) | 5.1 (5.1, 5.1) | 5.1 (5.1, 5.1) | 5.1 (5.1, 5.2) |
| **Concentricity (g/ml)** | 1 | 0.6 (0.5, 0.6) | 0.5 (0.5, 0.5) | 0.5 (0.5, 0.5) | 0.5 (0.5, 0.5) | 0.5 (0.5, 0.5) | 0.5 (0.5, 0.5) | 0.5 (0.5, 0.5) | 0.5 (0.5, 0.5) | 0.5 (0.5, 0.5) | 0.5 (0.5, 0.5) |
|  | 2 | 0.6 (0.5, 0.6) | 0.5 (0.5, 0.5) | 0.5 (0.5, 0.5) | 0.5 (0.5, 0.5) | 0.5 (0.5, 0.5) | 0.5 (0.5, 0.5) | 0.5 (0.5, 0.6) | 0.5 (0.5, 0.5) | 0.5 (0.5, 0.5) | 0.5 (0.5, 0.5) |
| **LV ejection fraction (%)** | 1 | 61.7 (61.2, 62.1) | 61.4 (61.0, 61.9) | 61.4 (60.9, 61.8) | 61.2 (60.8, 61.7) | 61.0 (60.6, 61.4) | 61.1 (60.7, 61.6) | 61.0 (60.5, 61.4) | 60.7 (60.3, 61.2) | 61.0 (60.6, 61.5) | 60.1 (59.7, 60.6) |
|  | 2 | 61.7 (61.2, 62.1) | 61.4 (61.0, 61.9) | 61.4 (61.0, 61.8) | 61.2 (60.8, 61.7) | 61.0 (60.6, 61.5) | 61.2 (60.7, 61.6) | 61.0 (60.5, 61.4) | 60.7 (60.3, 61.2) | 61.0 (60.6, 61.5) | 60.1 (59.7, 60.6) |
|  | **Men** | | | | | | | | | | |
| **Indexed LVEDV (ml/m^2^)** | 1 | 77.9 (76.5, 79.2) | 80.8 (79.5, 82.1) | 81.9 (80.6, 83.3) | 84.6 (83.3, 85.9) | 84.2 (82.9, 85.5) | 85.1 (83.8, 86.4) | 86.2 (84.8, 87.5) | 87.4 (86.1, 88.7) | 88.9 (87.6, 90.2) | 91.4 (90.1, 92.7) |
|  | 2 | 77.8 (76.5, 79.1) | 80.8 (79.5, 82.1) | 82.0 (80.7, 83.3) | 84.6 (83.3, 85.9) | 84.1 (82.8, 85.4) | 85.1 (83.8, 86.4) | 86.2 (84.9, 87.5) | 87.4 (86.1, 88.7) | 88.9 (87.6, 90.2) | 91.4 (90.1, 92.8) |
| **Indexed LV mass (g/m^2^)** | 1 | 48.1 (47.5, 48.8) | 49.0 (48.3, 49.7) | 48.8 (48.2, 49.5) | 50.3 (49.6, 51.0) | 50.2 (49.5, 50.9) | 50.2 (49.6, 50.9) | 50.6 (50.0, 51.3) | 51.9 (51.2, 52.6) | 51.7 (51.0, 52.4) | 53.4 (52.7, 54.1) |
|  | 2 | 48.0 (47.4, 48.7) | 48.9 (48.2, 49.5) | 48.9 (48.3, 49.6) | 50.3 (49.7, 51.0) | 50.1 (49.4, 50.8) | 50.3 (49.7, 51.0) | 50.8 (50.1, 51.5) | 51.9 (51.2, 52.5) | 51.7 (51.0, 52.3) | 53.4 (52.8, 54.1) |
| **Maximum LV wall thickness (mm)** | 1 | 7.6 (7.5, 7.7) | 7.6 (7.5, 7.6) | 7.4 (7.4, 7.5) | 7.6 (7.5, 7.6) | 7.5 (7.5, 7.6) | 7.5 (7.5, 7.6) | 7.5 (7.5, 7.6) | 7.6 (7.5, 7.6) | 7.5 (7.5, 7.6) | 7.7 (7.6, 7.7) |
|  | 2 | 7.6 (7.5, 7.6) | 7.5 (7.5, 7.6) | 7.5 (7.4, 7.5) | 7.6 (7.5, 7.6) | 7.5 (7.4, 7.6) | 7.5 (7.5, 7.6) | 7.6 (7.5, 7.6) | 7.6 (7.5, 7.6) | 7.5 (7.5, 7.6) | 7.7 (7.6, 7.7) |
| **Average LV wall thickness (mm)** | 1 | 6.0 (6.0, 6.1) | 6.0 (6.0, 6.1) | 6.0 (5.9, 6.0) | 6.0 (6.0, 6.1) | 6.0 (6.0, 6.1) | 6.0 (6.0, 6.1) | 6.0 (6.0, 6.1) | 6.1 (6.0, 6.1) | 6.0 (6.0, 6.1) | 6.1 (6.1, 6.2) |
|  | 2 | 6.0 (6.0, 6.1) | 6.0 (6.0, 6.1) | 6.0 (5.9, 6.0) | 6.0 (6.0, 6.1) | 6.0 (6.0, 6.1) | 6.0 (6.0, 6.1) | 6.0 (6.0, 6.1) | 6.1 (6.0, 6.1) | 6.0 (6.0, 6.1) | 6.1 (6.1, 6.2) |
| **Concentricity (g/ml)** | 1 | 0.6 (0.6, 0.6) | 0.6 (0.6, 0.6) | 0.6 (0.6, 0.6) | 0.6 (0.6, 0.6) | 0.6 (0.6, 0.6) | 0.6 (0.6, 0.6) | 0.6 (0.6, 0.6) | 0.6 (0.6, 0.6) | 0.6 (0.6, 0.6) | 0.6 (0.6, 0.6) |
|  | 2 | 0.6 (0.6, 0.6) | 0.6 (0.6, 0.6) | 0.6 (0.6, 0.6) | 0.6 (0.6, 0.6) | 0.6 (0.6, 0.6) | 0.6 (0.6, 0.6) | 0.6 (0.6, 0.6) | 0.6 (0.6, 0.6) | 0.6 (0.6, 0.6) | 0.6 (0.6, 0.6) |
| **LV ejection fraction (%)** | 1 | 58.4 (57.9, 59.0) | 58.4 (57.8, 58.9) | 58.3 (57.7, 58.8) | 57.6 (57.1, 58.2) | 58.4 (57.9, 59.0) | 57.6 (57.1, 58.2) | 57.9 (57.3, 58.5) | 57.7 (57.1, 58.2) | 57.6 (57.0, 58.1) | 57.4 (56.9, 58.0) |
|  | 2 | 58.4 (57.8, 59.0) | 58.4 (57.8, 58.9) | 58.3 (57.8, 58.9) | 57.6 (57.1, 58.2) | 58.4 (57.8, 58.9) | 57.7 (57.1, 58.2) | 57.9 (57.4, 58.5) | 57.7 (57.1, 58.2) | 57.6 (57.0, 58.1) | 57.4 (56.9, 58.0) |

Data as mean (95% CI).

**Model 1 (main model)**: Adjusted for wear duration, season, age, ethnicity, deprivation, smoking status, diabetes, statin medication, family history of CVD, family history of hypertension. Wall thickness outcomes, concentricity, and ejection fraction models additionally adjusted for body surface area.

**Model 2**: Adjusted for Model 1 plus systolic blood pressure.

# **Figure S1:** Flow of cohort selection


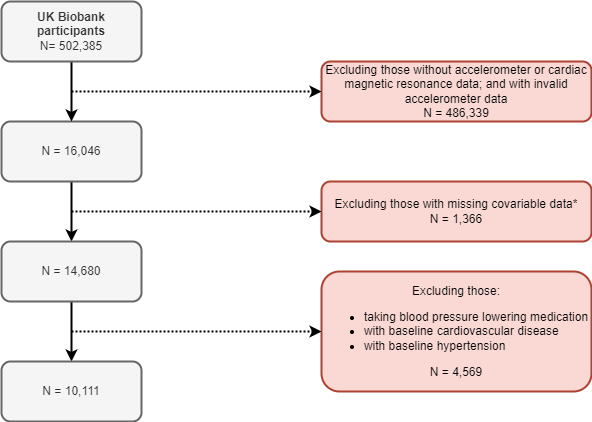


Prevalent CVD (cardiovascular disease) identified at baseline, or on any visit at or preceding the imaging assessments (defined as instance 0, 1 or 2), using the UK Biobank data-field (DF) 20002 with codes: 1067, 1074, 1075, 1076, 1079, 1588, 1591, 1592, 1492, 1087, 1081, 1082, 1083, 1086, 1491, 1583.

Prevalent hypertension identified at baseline, or on any visit at or preceding the imaging assessments (defined as instance 0, 1 or 2), using DF 20002 with codes: 1065, 1072.

Blood pressure lowering medications were identified using DF 20003 with codes: 1140888578, 1140866692, 1140866704, 1140866712, 1140866724, 1140866726, 1140866738, 1140866756, 1140866758, 1140866764, 1140866766, 1140866778, 1140866782, 1140866784, 1140866798, 1140866800, 1140866802, 1140866804, 1140909368, 1140879760, 1140879758, 1140879762, 1140879818, 1140879822, 1140879824, 1140879834, 1140879842, 1141145658, 1141145660, 1141145668, 1141152998, 1141153006, 1141156836, 1141166006, 1141171336, 1141171344, 1141193282, 1140866340, 1140860806, 1140860802, 1140860776, 1140860758, 1140860752, 1140860750, 1140860728, 1140860714, 1140860706, 1140860696, 1140860878, 1140860882, 1140860892, 1140860904, 1140860912, 1140860918, 1140888552, 1140888556, 1140888560, 1141167822, 1140860790, 1140860784, 1140860764, 1140860738, 1140860736, 1140864952, 1141151016, 1141172682, 1141187788, 1141201038, 1140864950, 1140866460, 1140866466, 1140861138, 1140861166, 1140861176, 1140861190, 1140861194, 1140861202, 1140861276, 1140861282, 1140866484, 1140866516, 1140866546, 1140866554, 1140866574, 1140861136, 1141152600, 1140861088, 1140861090, 1140861106, 1140861110, 1140861114, 1140861120, 1140861128, 1140861130, 1140888646, 1141167832, 1140879810, 1141153026, 1141153032.

* Covariable data was taken at instance 2 (imaging visit) where available and included: age (DF 21003), ethnicity (DF 21000), deprivation (DF 189), smoking (DF 20116), diabetes (DF 20002; codes 1220, 1223), statin treatment (DF 20003; codes 1140861922, 1140861970, 1140861958, 1141146234, 1141192410, 1140888648), family history of CVD (DF 20107; DF 20110), family history of hypertension (DF 20107; DF 20110), systolic blood pressure (DF 4080).

Full details on all DFs are searchable on the UK Biobank website <https://biobank.ndph.ox.ac.uk/ukb/search.cgi>

# **Figure S2:** Association between MVPA and heart rate during the scan

Values of MVPA are sex-specific within deciles medians; bars indicate 95% confidence intervals. Blue, men; pink, women.

P-values show the linear trend

Adjusted for wear duration, season, age, ethnicity, deprivation, smoking status, diabetes, statin medication, family history of CVD, family history of hypertension, body surface area.

# **Figure S3:** Association between MVPA and left ventricle parameters at ages 50, 60 and 70 years

Values of MVPA are sex-specific within deciles medians; bars indicate 95% confidence intervals.

P-values show the linear trend

Adjusted for wear duration, season, age, ethnicity, deprivation, smoking status, diabetes, statin medication, family history of CVD, family history of hypertension. Wall thickness outcomes, concentricity, and ejection fraction models additionally adjusted for body surface area.

# **Figure S4:** Association between total physical activity and left ventricle parameters

Values of total physical activity are sex-specific within deciles medians; bars indicate 95% confidence intervals. Blue, men; pink, women.

P-values show the linear trend

Adjusted for wear duration, season, age, ethnicity, deprivation, smoking status, diabetes, statin medication, family history of CVD, family history of hypertension. Wall thickness outcomes, concentricity, and ejection fraction models additionally adjusted for body surface area.

Total physical activity is measured as average daily acceleration in milligravity (*mg*) units, which provides a measure of the total amount of movement conducted over the average day. 1 *mg* has been identified as the minimum clinically important difference for inactive adults being associated with meaningful differences in mortality and cardiovascular outcomes.^1^ This difference is equivalent to adding a 5-minute brisk walk to daily physical activity patterns.^1^

[1] Rowlands A, Davies M, Dempsey P, Edwardson C, Razieh C, Yates T. Wrist-worn accelerometers: recommending ~1.0 mg as the minimum clinically important difference (MCID) in daily average acceleration for inactive adults. *British Journal of Sports Medicine. 2021;55(14):814-5.*

# **Figure S5:** Association between vigorous-intensity physical activity and left ventricle parameters

Values of VPA are sex-specific within deciles medians; bars indicate 95% confidence intervals. Blue, men; pink, women.

P-values show the linear trend

The proportion taking 0 mins/day of VPA corresponded to the distribution covered by deciles 1-5 in women and deciles 1-4 in men.

Adjusted for wear duration, season, age, ethnicity, deprivation, smoking status, diabetes, statin medication, family history of CVD, family history of hypertension. Wall thickness outcomes, concentricity, and ejection fraction models additionally adjusted for body surface area.
